# Improved Ultraviolet Radiation Film Dosimetry Using OrthoChromic OC-1 Film†

**DOI:** 10.1111/php.13364

**Published:** 2020-12-28

**Authors:** David Welch, David J. Brenner

**Affiliations:** Center for Radiological Research, Columbia University Irving Medical Center, New York, NY, USA

## Abstract

There is growing interest in far-UVC lighting, defined as wavelengths from 200 to 230 nm, because research has demonstrated these wavelengths to be an effective antimicrobial technology while posing a minimal hazard to human health. Far-UVC lighting is now being installed to directly irradiate spaces where humans are present, and it will be important to perform measurements to verify far-UVC lighting installations are operating within widely accepted exposure guidelines. In this work, we explore the use of a commercially available film, known as OrthoChromic OC-1, to measure ultraviolet radiation exposure. The film was tested with a variety of ultraviolet wavelengths and irradiance conditions, and the color change of the film was analyzed for increasing levels of radiant exposure. The film response extended over a dynamic range that was greater than the recommended exposure limits for far-UVC radiation so it can potentially be useful for health hazard monitoring. The spectrum of the incident ultraviolet radiation strongly affected the response of the film; therefore, for accurate measurements we recommend the measured spectrum match the spectrum used for calibration. Overall, dosimetry with this film provides a simple, accurate, and inexpensive method of quantifying ultraviolet radiation exposure that is suitable for far-UVC measurements.

## INTRODUCTION

Ultraviolet (UV) radiation is an established mechanism for disinfection of air and decontamination of surfaces (1–3). The current COVID-19 pandemic (4) has ushered UV radiation into the forefront as an intervention technology to help prevent the spread of this disease. Conventional germicidal UV lighting, which commonly uses low-pressure mercury lamps (LP Hg) principally emitting at about 254 nm, is currently used in a variety of settings with success; however, the inherent health hazards of this wavelength of light (5,6) limit its use to situations where humans are not directly exposed. An emerging approach to UV disinfection is the use of far-UVC lighting. Our group (7–14), along with many others (15–37), is exploring the safety and efficacy of this range of wavelengths. This far-UVC approach, which uses wavelengths in approximately the 200–230 nm range, has been demonstrated to have similar antimicrobial properties to conventional germicidal lighting but without the associated health hazards. This combination of efficacy and safety support far-UVC as a powerful tool for preventing the spread of disease.

Far-UVC lighting is beginning to be installed to directly irradiate human occupied areas. Occupational safety guidelines, such as those published by the American Conference of Governmental Industrial Hygienists (ACGIH) (38) and the International Commission on Non-Ionizing Radiation Protection (ICNIRP) (39), provide widely accepted safe exposure levels for UV lighting. These safe exposure limits are called threshold limit values (TLVs) by the ACGIH and exposure limits (ELs) by the ICNIRP, with the TLVs and ELs generally in agreement for values throughout the UV. The TLV (or EL) specifies the 8-hour exposure limit under which it is expected that humans may be repeatedly exposed without acute effects or risk of delayed effects. The TLV is wavelength dependent and limits for far-UVC exposure are higher than for conventional germicidal UV, for example, the TLV for 222 nm light is 23 mJ/cm^2^ while the TLV for 254 nm light is only 6 mJ/cm^2^. Verification that human exposure is kept below these limits is necessary for any installation of UV lighting.

Computer modeling of room lighting installations is common for design and planning purposes, and some of these programs are now being adapted to include far-UVC lighting fixtures. While modeling with these programs will provide useful data, a direct measurement of a far-UVC installation will still be necessary, just as these direct measurements are standard for installations which use conventional 254 nm UVC light (40). Irradiance and radiant exposure measurements at multiple locations throughout the irradiated volume will provide verification that the installation is operating as modeled. Repeating these measurements periodically after the initial installation can also verify continued operation as designed. These physical measurements can assure both the operators of the installations and the individuals using these spaces that safe exposure conditions are maintained.

There are limited techniques for measurement of UV irradiance and radiant exposure. One method is the use of calibrated radiometers or optical power meters which use a solid state sensor for measurements. These scientific instruments require a special sensor and electronic meter for measurements, and are used for benchtop laboratory measurements and also for in-field assessment of UV exposure. Their sizes vary, but even small models such as the Hamamatsu C9536/H9535-222 (Hamamatsu Photonics K.K., Japan) require a controller, cable, and sensor head and weigh about 400 grams. These electronic meters are also expensive, often on the order of thousands of US dollars each, and operation times can be limited by battery life when prolonged measurements are required away from a power source. As these are scientific instruments, periodic calibration is required for verification of accuracy. Furthermore, these devices are only capable of measuring at one location at a time, so completing an analysis of irradiance in multiple locations across a room or received by multiple individuals within the irradiated space is not easily accomplished. Another option for measurements of UV are calibrated spectrophotometers, which are also based on solid state measurement devices, but these suffer from many of the same drawbacks as power meters.

We have previously presented the use of unlaminated EBT3 Gafchromic film (Ashland Specialty Products) for monitoring UVC radiant exposure (41,42). That film was demonstrated to have a wavelength dependent response throughout the UVB and UVC regions. While EBT3 film is a useful tool, it has drawbacks that limit its utility. For instance, standard EBT3 radiochromic films, and therefore the unlaminated version of EBT3 previously tested, are designed to be analyzed using transmitted light. Changes in optical density therefore require measurement with a flatbed scanner operated in transmission mode or a densitometer. A related issue lies in the inherent dynamic range of unlaminated EBT3 with UVC wavelengths. As shown in our 2017 paper, for 222 nm light over half of the film net optical density response was achieved with only 1 mJ/cm^2^ of exposure and over 90% of the response was produced with only 10 mJ/cm^2^ (41). The safety concern for implementation of 222 nm far-UVC lighting within occupied spaces is a radiant exposure dose over the ACGIH TLV of 23 mJ/cm^2^ in an 8-hour period, so a useful response range that far exceeds that is preferable to assess hazards. Furthermore, if exposure limits for far-UVC wavelengths are increased in the future, which is possible given the evidence of diminished health hazards of these wavelengths (7–9,36,37), then a matching extended response range would be required.

We have identified a novel material for UVC dosimetry called OrthoChromic Film OC-1 (Orthochrome Inc., Hillsborough, NJ). This film, like other radiochromic films, was designed and marketed for use with ionizing radiation for radiotherapy quality assurance. We report here on the utility of this new film for UVC dosimetry with an emphasis on far-UVC exposure situations. This technology is especially important as far-UVC lighting expands in usage, and these fixtures are installed with the intent of directly exposing humans.

## MATERIALS AND METHODS

### Light Sources.

Four light sources were used to examine the film response to UV exposure. A krypton-bromine (KrBr) excimer lamp (High Current Electronics Institute, Tomsk, Russia) was used to emit at 207 nm. All experiments with the KrBr source included the addition of a custom bandpass filter (208NB6, Omega Optical, Brattleboro, VT), with a center wavelength of 208 nm and full width at half maximum of 6 nm, which essentially removed all but the dominant 207 nm emission peak. The second source used was a krypton-chlorine (KrCl) excimer lamp (High Current Electronics Institute, Tomsk, Russia) which emits principally at 222 nm; that lamp was used without any additional filtration. The third source was a filtered KrCl excimer lamp. We used a 12 W Ushio B1 module KrCl lamp (Ushio America, Cypress, CA) which includes an integrated custom filter to reduce the emissions outside the 222 nm peak. The transmission spectrum of the filter, obtained with the use of a deuterium lamp (63945, Newport, Irvine, CA), is provided in the Supporting Information (Figure S1). The fourth light source used for film testing was a low-pressure mercury lamp (LP Hg) (Mineralight XX-15S, UVP, Upland, CA) which was principally emitting 254 nm light.

### Light Characterization.

**Figure 1.**
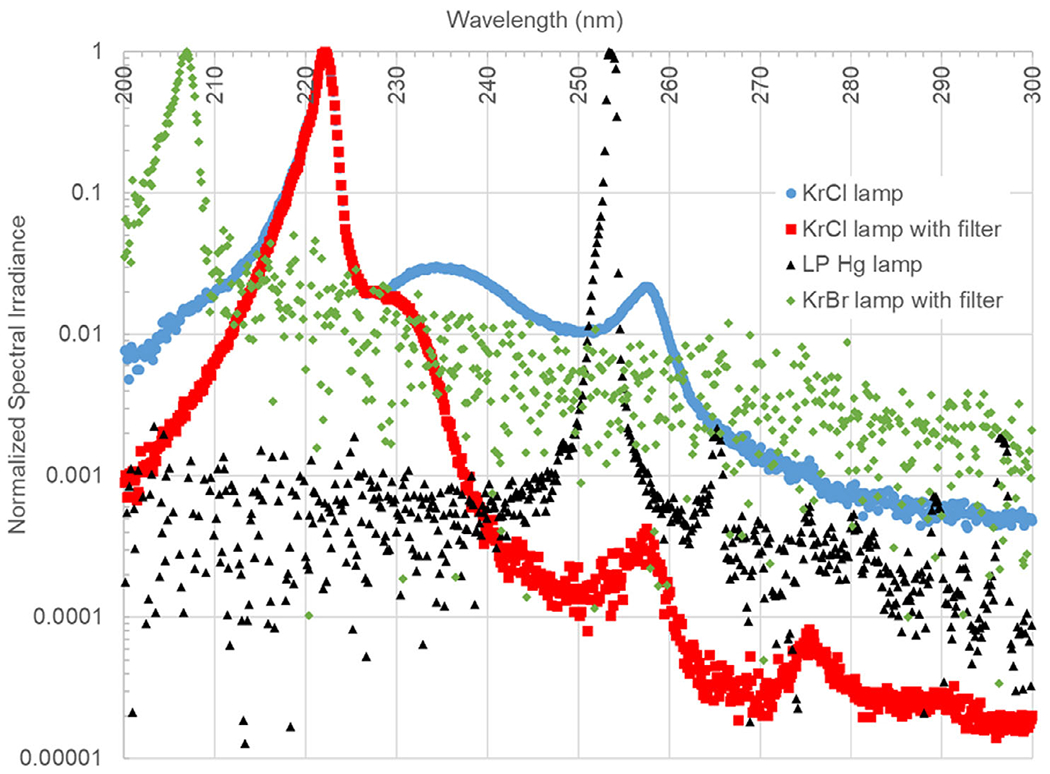
The normalized spectral irradiance of the four UV sources tested in this study. The LP Hg lamp, KrBr lamp with filter, and KrCl lamp without filter plots were all directly captured with the spectrometer. The noise outside of the peak regions for the LP Hg and KrBr lamps is the noise inherent to the device. The KrCl lamp with filter plot has been generated by multiplying the unfiltered KrCl spectrum with the transmission of the filter in order to better illustrate the filtered spectrum since it would otherwise not be visible in the device noise.

An AvaSpec-ULS4096CL-EVO spectrometer (Avantes Inc., Louisville, CO) calibrated to measure absolute irradiance was used to obtain the spectrum of all sources. The normalized spectral irradiance for each of the lamp configurations used in this study is shown in Fig. 1. The spectral irradiance of the filtered KrCl lamp was plotted by multiplying the unfiltered KrCl spectrum by the transmission of the custom filter. This approach for obtaining the filtered KrCl spectrum was required because the addition of a filter to the KrCl lamp decreased the emissions outside of the 222 nm peak below detectable levels for the spectrometer. The noise floor of the spectrometer is evident in the traces for the filtered KrBr lamp and the LP Hg lamp outside of the primary peak area; the nonpeak emissions on these traces are merely noise and not at levels representative of the spectral contributions beyond the principle peak. The noise floor appears at different levels because the spectra have been normalized to the peak irradiance which varied between sources. In addition to the calibrated spectrometer, a calibrated Hamamatsu UV Power Meter (C9536/H9535-222, Hamamatsu Photonics K.K., Japan) was also used to verify the irradiance values during film exposures.

### UV Sensitive Film.

The film product used in this study is OrthoChromic Film OC-1 (Orthochrome Inc., Hillsborough, NJ). OC-1 film is marketed as a tool for radiation therapy measurements. The flexible film is 155 μm thick, consisting of a 30 μm active coating on a 125 μm white polyester base. Unlike the unlaminated EBT3 film used in our previous study, the OC-1 film is fully waterproof. The active region must be oriented toward the UV source during measurements since the polyester layer is opaque.

### Film Analysis.

We utilized an Epson Perfection V700 photo flatbed scanner (Epson, Suwa, NGN, Japan) for quantification of the color of each film. The scanner was operated in reflection mode and captured 48 bit RGB TIFF images with all color correction factors turned off. MATLAB version R2019a (Mathworks, Natick, MA) with the image processing toolbox was used for analysis, with each of the three color channels processed independently. The color density (CD) of each pixel value was calculated with the equation.

(1) $${\text{CD}=-\text{log}}_{10}\left(\frac{\text{pixel value}}{65536}\right)$$

and the net color density calculated as.

(2) $$\text{netCD}=\text{CD}-{\text{CD}}_{\text{unexposed}}=d_{x}\left(D\right),$$

with the CD_unexposed_ being the *CD* for an unexposed piece of film and *d*_*x*_*(D)* being the response for a given radiant exposure dose (*D*). Data for each exposure condition were matched to a fitting function with the form.

(3) $$d_{x}\left(D\right)=\frac{a+bD}{D+c}$$

where *a*, *b*, and *c* are constants. The fitting function was optimized in MATLAB using the curve fitting tool to minimize the squared difference between the experimental data and the fit equation.

## RESULTS

### Film Color Change With Increasing Radiant Exposure

**Figure 2.**
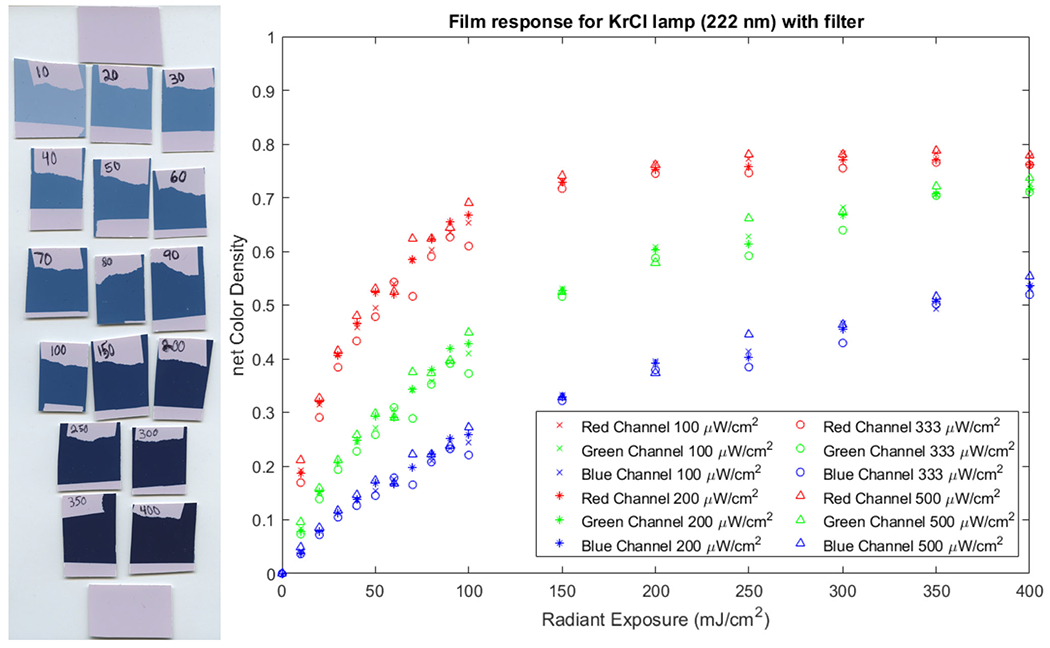
The color response of the OC-1 film with increasing radiant exposure to a filtered KrCl lamp is shown in the image on the left. This set of films were all exposed using an irradiance of 100 μW/cm^2^. The net color density for increasing radiant exposures is plotted on the right. Four different irradiance values were tested for each radiant exposure dose.

The visible color change of the film upon exposure to the filtered KrCl lamp emitting at 222 nm is shown in Fig. 2. Exposures for this set of films used an irradiance of 100 μW/cm^2^, and the total radiant exposure ranged from 10 mJ/cm^2^ to 400 mJ/cm^2^. The radiant exposure for each film was recorded on a region which was covered with tape during the exposure. Unexposed films are included at the top and bottom of the figure for reference.

### Test of Irradiance Dependence

The film response was examined using four different irradiance values: 100 μW/cm^2^, 200 μW/cm^2^, 333 μW/cm^2^, and 500 μW/cm^2^. Radiant exposures ranging from 10 mJ/cm^2^ to 400 mJ/cm^2^ were administered for each irradiance. Figure 2 shows the dose-dependent response plotted for each color channel for all irradiance values used.

### Test of Lamp Spectrum Dependence

**Figure 3.**
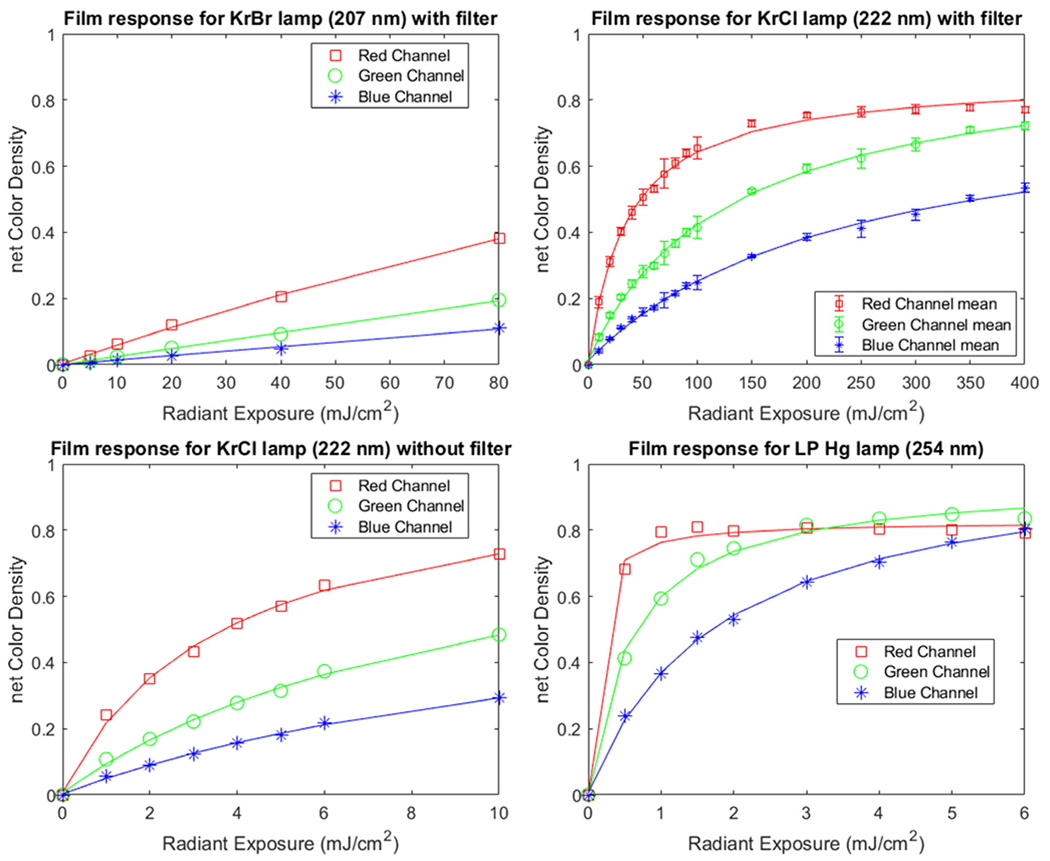
The film response is plotted for increasing radiant exposures for the four different lamp and filter combinations tested in this study. The red, green, and blue channel color density response is shown for each film. The calculated fit line matching the form of Equation 3 is plotted on top of each color channel. Data points for the filtered KrCl lamp, shown in the top right plot in this figure, are the mean of the data points shown in Fig. 2, with error bars representing standard deviation.

Film responses for each of the four spectrums tested in this study are shown in Fig. 3. The response curves show a large dependence on the wavelength of the incident radiation. The 254 nm LP Hg lamp showed the highest change in net color density for a given radiant exposure. Saturation in the red channel was seen after only ~ 2 mJ/cm^2^ of LP Hg exposure and represents the upper range over which the red color response could be used for dosimetry. The smallest change in net color density for a given radiant exposure with the examined sources was observed for the 207 nm KrBr source. The response for a filtered 222 nm KrCl lamp is also observed to differ from the unfiltered KrCl lamp response. The filtered 222 nm response curve was produced using the average of the four different irradiance values tested in this study. All points for each color channel for each exposure condition were optimized to fit to Equation 3, and each fit line produced an R-squared value of greater than 0.993. Fit equation coefficients and R-squared values for each of the fit lines plotted are included in Table 1.

## DISCUSSION

We tested OrthoChromic OC-1 film using a variety of UVC spectra, irradiances, and radiant exposures to examine its functionality. Through these tests, we observed that the film response is highly dependent on the spectrum of radiation used during the exposure. This finding aligns well with our previous work with radiochromic film for UVC dosimetry (41). The magnitude of this dependence is highlighted when comparing the KrCl lamp with and without filtration (Fig. 3). The sensitivity of this film is notably higher for exposures in the wavelength range of 254 nm than it is for 222 nm light. Even though the unfiltered KrCl lamp only has a small component outside of the peak emission, those emissions dramatically change the film response. We can conclude that the most important consideration when using this film is that the film be calibrated using the exact lamp and filter combination that is to later be evaluated.

We exposed this film using different irradiance values to examine the potential for dose-rate dependence. OrthoChromic OC-1 films, like other radiochromic films, are marketed as being dose-rate independent, so we expected this behavior to hold for UVC exposures as well. Over the range of irradiances used in this work, we did not observe any difference in film response. Some radiant exposure doses do show a higher standard deviation, but this can likely be attributed to variations in the irradiance from the lamp at close distances, which was required for higher irradiance exposures, and small differences in the placement of the film during the calibration exposures.

The dynamic range of film for UVC dosimetry is important for its practical use. Our previous work with unlaminated EBT3 film found that film to have a somewhat limited dynamic range, even with 222 nm light. Our results here show the dynamic range of OC-1 film to 222 nm light from a filtered KrCl source to extend well above the ACGIH TLV before any saturation occurs. The dynamic range for 207 nm light is even larger. Many situations for personal monitoring of exposure would want to sample over a long period of time to get an average dose received over an extended period. The large useful range of this film makes those measurements possible.

Overall, the OrthoChromic OC-1 film tested here has many qualities, in terms of film response, that make it an excellent choice for far-UVC dosimetry. Additional advantages occur due to the physical composition of this film since it includes a white polyester backing layer. This backing layer requires analysis of the film to be performed on reflected light instead of transmitted light. Using reflected color change enables one to more easily evaluate the film since backlighting is not required. The color change can potentially be viewed by an individual and then compared against a color scale to obtain an approximate exposure dose without computer image analysis. Obtaining results with reflected light also allows for the film to be analyzed with a smartphone. The procedure for analysis could then involve using a smartphone camera to obtain a raw color image (without any color corrections, similar to the methods used here), normalize the image to correct for lighting and shadowing across the film, and then analyze the color density change using a known calibration curve. With a smartphone to analyze the film, it is then straightforward to have the user record the length of the exposure and then calculate the average irradiance received on the film. This value could then be compared to the TLV and also used to advise on the safety or the efficacy of the installed UVC lighting. Furthermore, a smartphone could log the results for a user over time as well as report results to a central database for larger-scale analysis. Logging the location and time of exposures with the smartphone used for analysis would also help construct a map of UV irradiance throughout a space.

The dynamic range of this film could be extended for any wavelength showing a response by adding a partially absorbing material on top of the active layer. This layer would effectively reduce the magnitude of the irradiance reaching the active layer, so a smaller color change would occur for a given incident exposure. However, since the absorption qualities of the filtering material are also likely highly dependent on wavelength, it would of course be necessary to calibrate the film dose response with this added filter present.

Our previous work with unlaminated EBT3 film utilized the red channel of the film for analysis (41). As evident in all of the response curves in Fig. 3, and similar to other radiochromic films, there is a dose-dependent response for each of the colors. The red channel of this film offers the most sensitivity. However, the red channel also saturates the fastest. The green channel net color density range is almost as large as in the red channel, but the slope is not as drastic so a much larger radiant exposure is required before saturation. In order to have the largest dynamic range, the use of the green channel for film analysis could prove most useful. Differences in the response curve shapes shown in Fig. 3 are due to the portion of the response curve being used. At lower doses, the relationship maintains an almost linear relationship even in the red color channel which shows the highest sensitivity. The general shape of the response curve appears identical regardless of the spectrum, but the magnitude of the color response has a clear wavelength dependence.

While not examined methodically in this work, this film was found to be sensitive to exposure to UV wavelengths beyond the UVC. Specifically, pieces of this film were exposed to sunlight, which contains UVA and UVB light, and exhibited a color response. It is therefore recommended that exposure to sunlight or any other significant UV source be avoided when using this film to measure UVC exposures. Similarly, the manufacturer of OrthoChromic OC-1 advertises that indoor ambient light can slowly darken the film, so pieces should be shielded when not being used. As with other film for radiation dosimetry, the color change of the film will grow slowly over time following exposure, so it is best to measure for color change within 48 hours following exposure.

We have found OrthoChromic OC-1 film to be a promising tool for UVC dosimetry. The film is relatively inexpensive so many measurements can be made with minimal cost. The film can be cut to a desired shape or size. Small pieces of film can easily be affixed to a surface to measure irradiance upon that surface. A piece can also be attached to a person through the use of a badge with film affixed to it or even by directly attaching the film onto clothing, so personal dosimetry is simple and unobtrusive. Large pieces can also be exposed to verify the 2D irradiance values across an exposure field with high resolution. Compared to our previous work with unlaminated EBT3 film, OrthoChromic OC-1 film has a larger dynamic range which makes it better suited for measurements relevant for potential human exposures with far-UVC lighting. Additionally, film analysis using reflected color measurement instead of optical density measurement provides more options for dose readout and increases the ease of use of this technology. Overall, the film dosimetry approach described here can help assess the safety of far-UVC installations and can also prove useful for any other UVC dosimetry application.

## Supplementary Material

Figure S1. The transmittance of the filter used with the KrCl lamp is plotted on both a linear scale (left) and a log scale (right).

## Figures and Tables

**Table 1.** The coefficients and R-squared values for each spectrum tested are provided for the red, green, and blue color channels. The coefficients were determined by fitting the data shown in Fig. 3 using Equation 3

Lamp	Color	A	B	C	*R* ^2^
KrBr filtered (207 nm)	Red	0.6173	1.961	333.9	0.9985
	Green	0.0002857	71.76	2.962e + 04	0.9985
	Blue	4.816e-07	20.1	1.498e + 04	0.9936
KrCl (222 nm)	Red	0.02809	0.9954	3.701	0.9969
	Green	0.07502	0.9599	10.01	0.9971
	Blue	0.04095	0.7008	14.05	0.9980
KrCl filtered (222 nm)	Red	4e-05	0.87	35.33	0.9967
	Green	1.684	0.9515	128.5	0.9984
	Blue	2.733	0.8245	237	0.9974
LP Hg (254 nm)	Red	5.502e-05	0.8257	0.08174	0.9944
	Green	2.304e-14	0.9525	0.5889	0.9954
	Blue	0.01114	1.039	1.845	0.9989
