# Supplementary material for: Improved Ultraviolet Radiation Film Dosimetry Using OrthoChromic OC-1 Film
Source: Photochem Photobiol. Author manuscript; Available in PMC 2021 Oct 26. (PMC8547612; doi:10.1111/php.13364)
Supplement: Figure S1. — The transmittance of the filter used with the KrCl lamp is plotted on both a linear scale (left) and a log scale (right). [file NIHMS1726251-supplement-supplementary_material.pdf]

SUPPORTING INFORMATION

Improved Ultraviolet Radiation Film Dosimetry Using OrthoChromic OC-1 Film

David Welch*, David J. Brenner

Center for Radiological Research, Columbia University Irving Medical Center, New York, NY

**Corresponding author email: dw2600@cumc.columbia.edu* (David Welch)

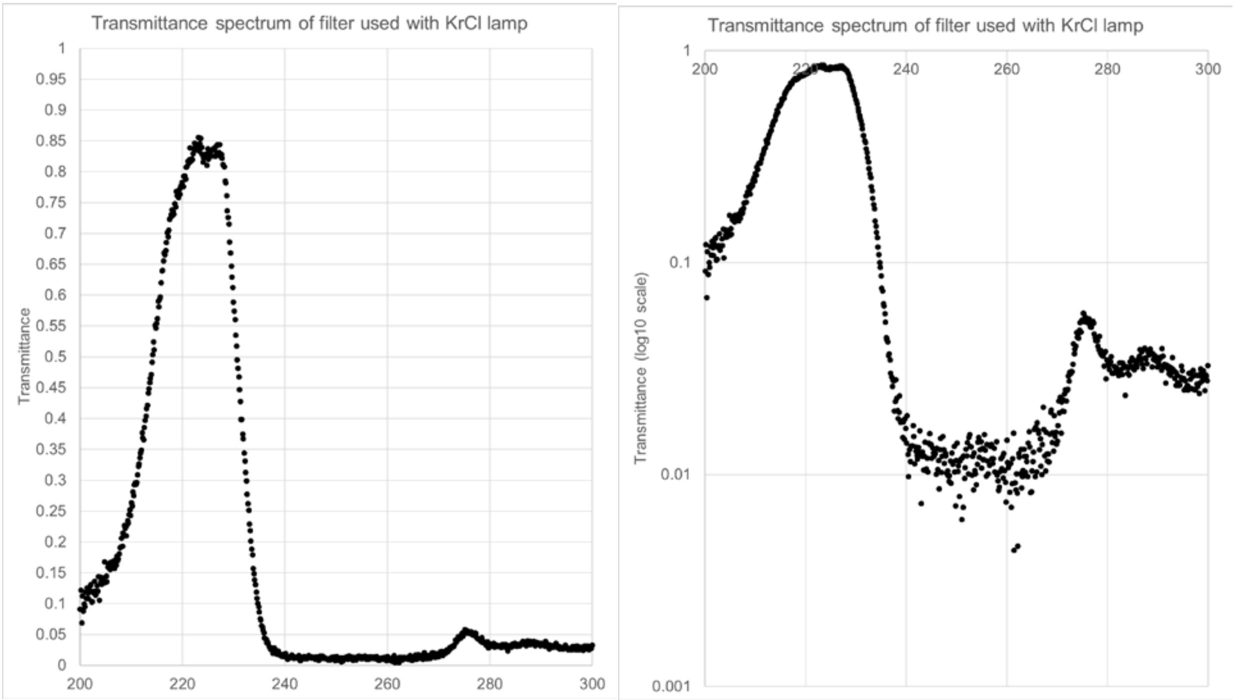

Figure S1. The transmittance of the filter used with the KrCl lamp is plotted on both a linear scale (left) and a log scale (right). The transmittance values for the filter were multiplied by the unfiltered KrCl spectrum to produce the filtered KrCl spectrum plotted in Fig. 1.
